# Understanding the influence of lipid bilayers and ligand molecules in determining the conformational dynamics of somatostatin receptor 2

**DOI:** 10.1038/s41598-021-87422-5

**Published:** 2021-04-07

**Authors:** Santhosh Kumar Nagarajan, Sathya Babu, Seema A. Kulkarni, Aanand Vadivelu, Panneer Devaraju, Honglae Sohn, Thirumurthy Madhavan

**Affiliations:** 1 Computational Biology Lab, Department of Genetic Engineering, School of Bioengineering, SRM Institute of Science and Technology, SRM Nagar, Kattankulathur, Chennai, 603203 India; 2 Department of Food and Process Engineering, School of Bioengineering, SRM Institute of Science and Technology, SRM Nagar, Kattankulathur, Chennai, 603203 India; 3 Unit of Vector Biology and Control, ICMR-Vector Control Research Centre, Indian Council of Medical Research (ICMR), Puducherry, India; 4 Department of Chemistry and Department of Carbon Materials, Chosun University, Gwangju, South Korea

**Keywords:** Computational biophysics, Membrane biophysics, Computational models, Protein structure predictions

## Abstract

Somatostatin receptor 2 (SSTR2) is a G-protein coupled receptor (GPCR) that controls numerous cellular processes including cell-to-cell signaling. In this study, we report how the lipid and ligand molecules influence the conformational dynamics of the membrane-bound SSTR2. Molecular simulations of different holo and apoenzyme complexes of SSTR2 in the presence and absence of a lipid bilayer were performed, observed, and correlated with previously reported studies. We identified the important SSTR2 residues that take part in the formation of the SSTR2-ligand complex. On analyzing the molecular simulation trajectories, we identified that the residue D3.32 is crucial in determining the bioactive conformation of SSTR2 ligands in the binding site. Based on the results, we suggest that designing a novel SSTR2 ligand with an H-bond donor group at the R1 position, and hydrophobic groups at R2 and R3 might have higher activity and SSTR2-selectivity. We analyzed the simulated systems to identify other important structural features involved in SSTR2-ligand binding and to observe the different conformational changes that occur in the protein after the ligand binding. Additionally, we studied the conformational dynamics of N- and C-terminal regions of SSTR2 in the presence and absence of the lipid bilayer. Both the systems were compared to understand the influence of lipid molecules in the formation of secondary structural domains by these extracellular regions. The comparative study revealed that the secondary structural elements formed by C-terminal residues in presence of lipid molecules is crucial for the functioning of SSTR2. Our study results highlight the structural complexities involved in the functioning of SSTR upon binding with the ligands in the presence and absence of lipid bilayer, which is essential for designing novel drug targets.

## Introduction

G-protein coupled receptors form the most diverse and largest group of proteins in the human proteome^1^. They control a varied series of signaling events inside the cell as feedback to various extracellular stimuli caused by enzymes, hormones, ions, neurotransmitters, photons etc^2^. Hence, GPCR-related signaling cascades have been involved in the pathophysiology of various human diseases, like neurological, gastrointestinal, respiratory, and cardiovascular disorders. It is important to note that ~ 30–40% of the prescribed drugs in the market target GPCRs^3^. Thanks to the present-day advancements in GPCR structural biology and pharmacology, GPCR drug discovery has been the most sought out target in the current pharmaceutical world. Understanding the relation between GPCR structure and function is essential for solving the complexities involved in GPCR-mediated cell signaling and thus contributes to the discovery of novel GPCR-related drugs. Every GPCR has a different mode of cellular communication, despite having similar structural domains like the highly conserved E/DRY motif and seven trans-membrane helices^4,5^. Thus, it is crucial to understand the structural changes that occur in domains and the ligand-binding sites during the ligand binding. In this study, we have studied the conformational dynamics of different structural domains of Somatostatin receptor 2 (SSTR2).

SSTRs belong to the GPCR superfamily, that are ubiquitous throughout the human body. The native ligand of the somatostatin receptors is a peptide hormone, known as Somatostatin (SST) or Somatotrophin release-inhibiting factor (SRIF). Together, they control an expansive range of biological events which primarily includes inhibiting the function of SST-related organs and cells. SSTR2, one of the five SSTR subtypes, is known to regulate the inhibition of acid and chloride production in the parietal cells and colonic epithelial cells, respectively^6^. SSTR2 expression in the basal ganglia suggests its possible involvement in the function of mucosa and the flow of blood^7^. As it is expressed extensively in the human peripheral and central nervous systems, it is regarded as a therapeutic target in treating medulloblastoma^8^. In periodontal cells and tissues, SSTR2 regulates the proinflammatory, microbial and obesity-related signals^9^. The binding of an agonist with SSTR2, either a natural ligand or a mimicking analog, initiates an intracellular signaling cascade which leads to inhibition of growth, synthesis of hormones, and apoptosis. Thus, it is imperative to understand the structural factors that are responsible for the formation of the SSTR-ligand complex. The structural information could clarify the molecular mechanism underlying the SSTR-related signaling. The results of the study could be useful in designing and developing various drugs to treat various SSTR2 related disorders. This study is part of an extensive project, to characterize the structural features responsible for the biological activity of SSTRs. We have already reported structural characterization studies of SSTR1^10^, SSTR2^11^, and SSTR5^12^ and this study is an extension of the structural characterization study of SSTR2^11^. In this study, we report the conformational understanding of the different structural domains of SSTR2 using molecular simulation, in the presence and absence of lipid and ligand molecules.

## Results

### Constructing reliable membrane-bound SSTR2 models

**Figure 1:**
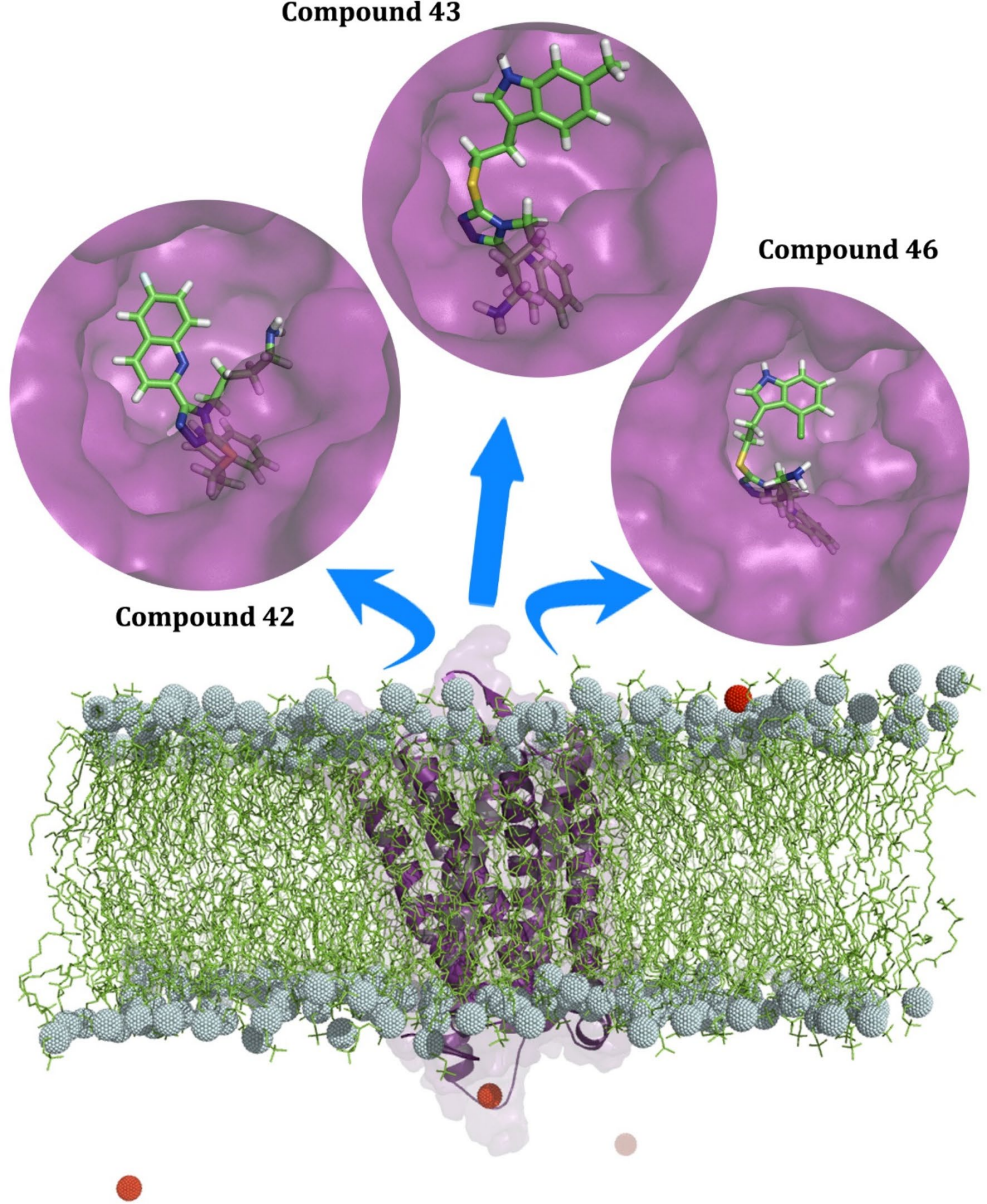
The figure represents the SSTR2 homology model embedded in a DPPC lipid bilayer. SSTR2 homology model is displayed as a purple colored cartoon representation. Lipid acyl chains and head phosphorous atoms are represented as green lines and cyan spheres, respectively. SSTR2 ligand molecules residing in SSTR2 binding site are represented in the circles. Ion molecules added in the system to neutralize the system charge are represented as red spheres. Figure generated using PyMOL 1.3 (http://www.pymol.org).

SSTR2 homology model, model **03**, was selected after model validation and optimization using Sybyl biomacromolecule preparation module. Molecular docking of the SSTR2 ligand dataset was performed and the corresponding protein–ligand complexes were saved. SSTR2-ligand complexes with ligands are embedded in lipid bilayer made up of DPPC molecules. In addition, a protein-lipid system with SSTR2 apoenzyme was also built to correlate with holoenzymes. Figure 1 represents the holoenzyme-lipid bilayer systems before simulation. We analyzed the potential energy of the systems over the simulation time to check whether the system stabilized energetically. The values equilibrated around − 2.84 × 10^+06^ kJ mol^−1^, − 2.86 × 10^+06^ kJ mol^−1^ and − 2.86 × 10^+06^ kJ mol^−1^ for systems with the compounds **42**, **43,** and **46,** respectively. To validate the reliability of these simulated systems, parameters of the bilayer including deuterium order parameters, the density of different simulated components of the system, mean square displacement, area per lipid were studied. Initially, the area per lipid (APL) of each lipid layer over simulated time was calculated using GridMAT-MD. The algorithm reads the GROMACS trajectory file and estimates the average thickness of the bilayer over the simulated time. Average APL of lipids in both layers of the systems with compounds **42**, **43** and **46** were 63.51 Å^2^, 65.77 Å^2^, and 66.82 Å^2^, respectively, which is near the experimentally calculated APL of a DPPC molecule (62.90 and 64.30 nm^2^ at 323 K)^13–16^. We plotted the bilayer thickness of various simulation components using the distribution of electron density profiles of the component. The density plots are represented in Figure S1 and we have observed that the densities of the simulation components were comparable to the corresponding experimental values (3.78 to 3.86 nm at 323 K^13–15^). Figure S2 represents the plots of order parameters for each system against the renumbered atoms. The plots were validated to check whether the membrane has successfully entered the gel phase or not during the simulation. Additionally, the mean square displacement (msd) of lipids along the plane is also estimated by keeping phosphorus atoms present in the head group as reference points. After calculation, msd of systems with **42**, **43** and **46** were observed as 0.00905 × 10^–5^ cm^−2^/s, 0.00871 × 10^–5^ cm^−2^/s and 0.01000 × 10^–5^ cm^−2^/s, respectively.

### Comparative study reveals the importance of lipid bilayer in system equilibration

**Figure 2:**
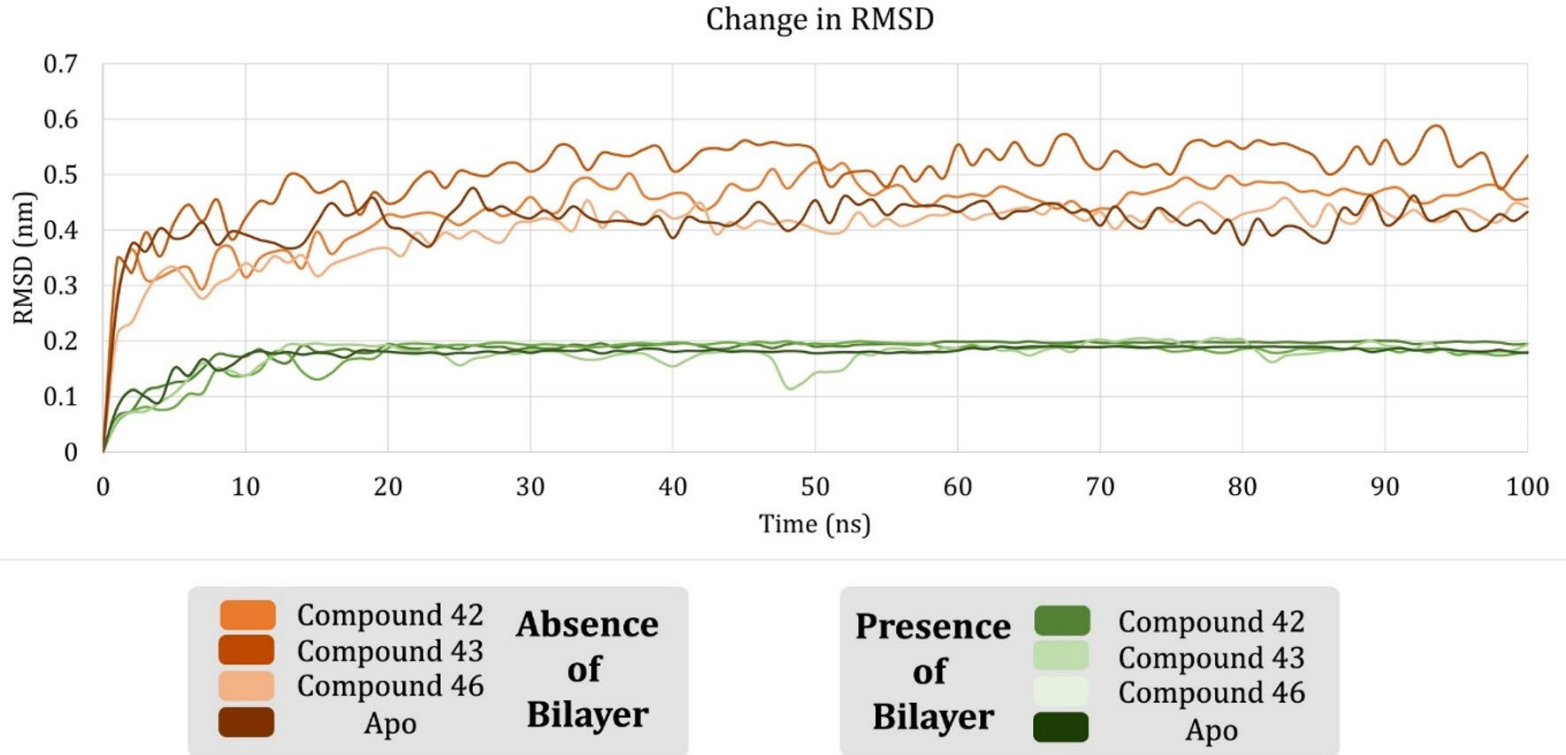
Comparing the time evolution plots of the RMSD of backbone Cα atoms in the presence and absence of a lipid bilayer. The systems showed better equilibration in the presence than in the absence of lipids.

Simulation trajectories were studied to understand the dynamics of protein over time and position of the ligand in the binding site. The observations from the trajectories were correlated with the simulations of the same complexes in absence of a bilayer^11^. We compared the change in RMSD of the 4 complexes in the presence and absence of the lipid bilayer (Fig. 2) and observed salient differences. Average *RMSD* (*RMSD*_*avg*_) of the complexes were calculated and analyzed along with the *RMSD*_*avg*_ of apoprotein simulation. In presence of a bilayer, complexes with compounds **42**, **43**, and **46** showed *RMSD*_*avg*_ of 0.1842 nm, 0.1752 nm, 0.1716 nm with a standard deviation (SD) of 0.030 nm, 0.034 nm, 0.033 nm, respectively. *RMSD*_*avg*_ of apoprotein over time was calculated (0.1760 nm) and compared with holo complexes. While correlating these RMSD values with the previous reports, we observed that in the absence of a bilayer, the backbone Cα atoms showed significantly lesser deviation. All the complexes, in both presence and absence of lipids, equilibrated after an initial spike in the RMSD. From the graph, it is prominent that the systems in presence of the bilayer showed better equilibration than the systems in absence of the lipid bilayer, and they equilibrated below 0.200 nm. Whereas, in absence of the bilayer, the system equilibrated well above 0.200 nm, almost as double the distance as in presence of the bilayer. This initial observation signifies the importance of a lipid bilayer in forming better equilibrated SSTR2 apo and holoenzyme systems. To further understand this phenomenon, we analyzed the change in the radius of gyration (*R*_*g*_) of SSTR2 backbone Cα atoms against time. We observed that the change in *R*_*g*_ in each complex is centered around 2.5 nm. Compounds **S2-42**, **S2-43,** and **S2-46** scored average *R*_*g*_ values of ~ 0.22 nm, 0.26 nm, and 0.27 nm, respectively. Values of *R*_*g*_ did not go beyond 0.29 nm, suggesting that the systems stayed inside the simulation box. Change in *R*_*g*_ of SSTR2 apo system against time was plotted to compare them with holo complexes. Apo showed a similar deviation to the complex systems (~ 0.25 nm) and considered negligible as the deviation was very minimal (~ 0.05 nm). Figure S3 represents the comparison of changes in *R*_*g*_ between apo and holoenzymes of SSTR2.

**Figure 3:**
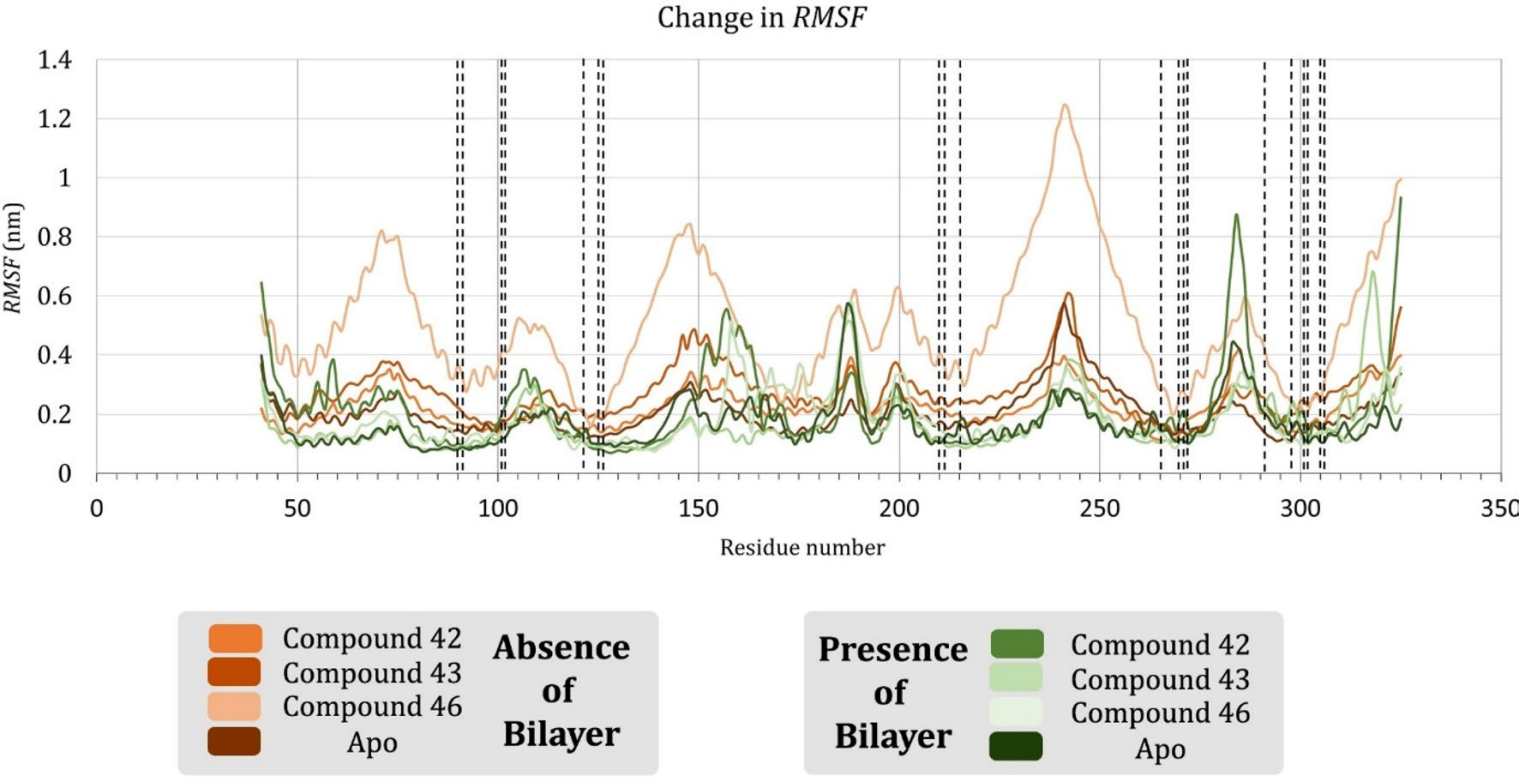
Comparing time evolution plots of the RMSF of SSTR2 amino acid residues in the presence and absence of a lipid bilayer. SSTR2 showed lesser fluctuation throughout the simulation than in the absence of lipids.

We plotted the change in *RMSF* of amino acid residues of the protein structure over the simulation time. After calculating the *RMSF*_*avg*_ of SSTR2 residues, the stable and fluctuating residues were defined. *RMSF*_*avg*_ for complexes with compounds **S2-42**, **S2-43** and **S2-46** were 0.2124 nm, 0.1669 nm, and 0.1837 nm, respectively. Residues of the apo system showed an *RMSF*_*avg*_ of 0.1687 nm. Residues L2.44, I2.43, A2.47, L2.46, D2.50, V3.45, E2.51, M3.46, I3.48, D3.49, and S3.47 were identified to be stable. Of these residues, V3.45, M3.46, S3.47, I3.48, and D3.49 were found to be stable in the ligand complexes than apo. In other words, in the apo system, the above-mentioned residues fluctuated above *RMSF*_*avg*_ and found to be stable in systems in the presence of a ligand. Residues fluctuating beyond the *RMSF*_*avg*_ were identified and most of them were found to localize either in loops or in terminal domains. Figure 3 represent the change in *RMSF* of SSTR2 residues in the presence and absence of lipid bilayer over simulation time. For example, residue L40 present in the N-terminal, residues N186, S185, Q187, G189, and W188 of ECL2, V242 of ICL3 and A283, I284, S285, and P286 of ECL3 were fluctuating more than *RMSF*_*avg*_. Residues like M6.61, T7.28, and P7.29 were also fluctuating more than *RMSF*_*avg*_ that do not belong in a loop or terminal. However, these residues are present adjacent to loops as they are the initial residues of the respective helices. Residues L40, V242, T7.28, P7.29, and N8.56 were stable in apo systems than in the ligand complex systems. On analyzing these results with the previously reported results^11^, the residues in presence of lipids were less fluctuating and more consistent throughout the simulation. Especially, the difference in the case of compound **42** can be clearly observed from the graph.

### Residues playing major role in ligand binding

**Figure 4:**
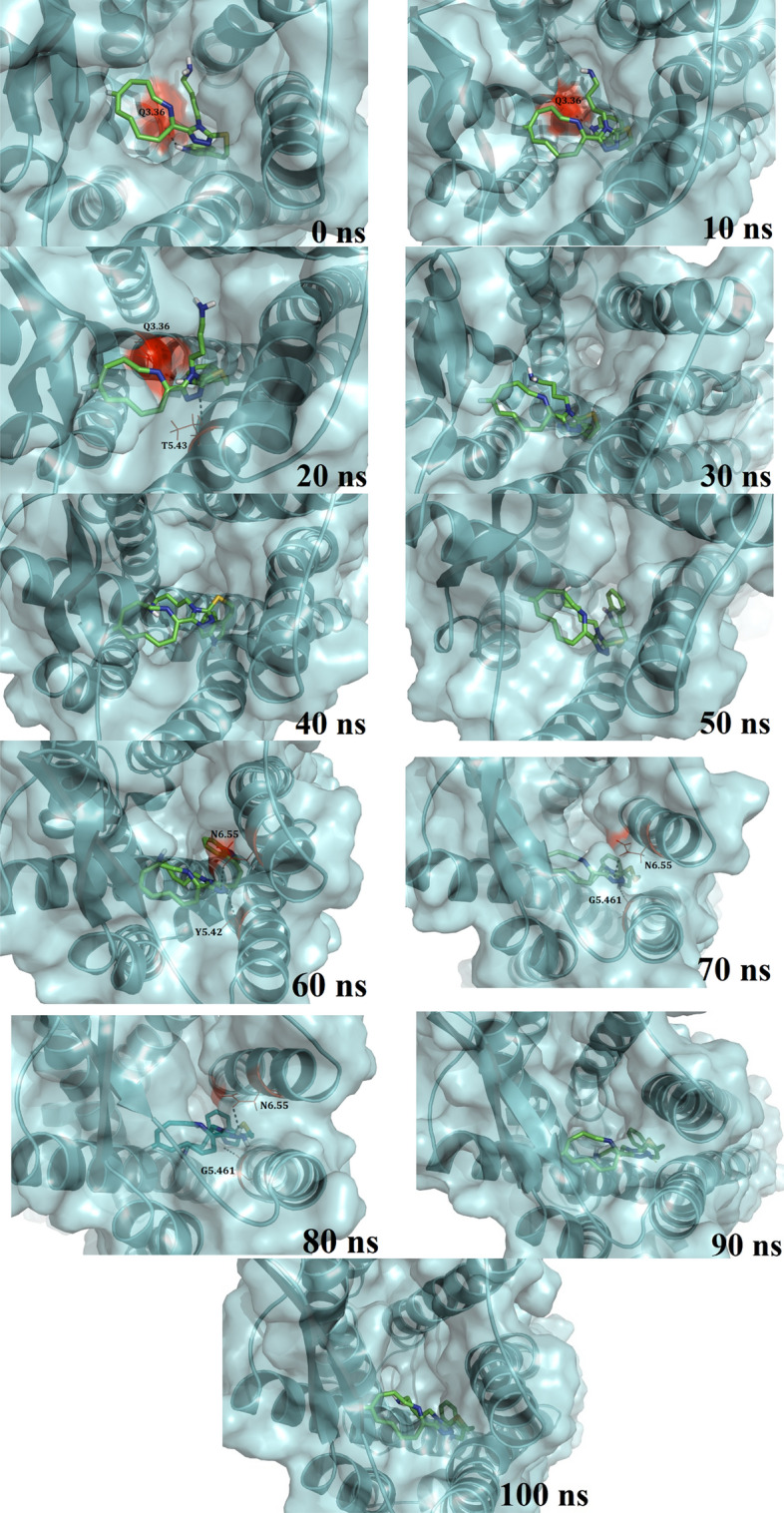
Time evolution of ligand bioactive conformation of compound 42 inside SSTR2 binding site. Figure generated using PyMOL 1.3 (http://www.pymol.org).

MD trajectories of the systems were extracted from each simulated system and snapshots at different times were taken for structural analysis. In the previous study, we have identified that the residues D2.50, F2.53, Q2.63, A2.65, D3.32, N3.35, Q3.36, I5.40, T5.43, G5.461, F6.44, W6.48, F6.51, Y6.52, N6.55, K7.31, V7.38, T7.41, Y7.42, N7.45, and S7.46 could be crucial in forming the SSTR2 binding site^11^. Along with these residues, other important SSTR2 residues that are reported in the literature as crucial for the bioactivity of ligands that target SSTR2 are carefully examined. For instance, Kaupmann et al.^17^ reported that residues of SSTR2 TM helices VI and VII, especially K7.31 and S7.46, are key determinants of the biological activity of SSTR2 ligands. K7.31 forms an integral part of the proposed binding site and was identified to be the most fluctuating binding site residue in both the apo and complex systems. Other than K7.31, residues like A2.65 and V7.38 also fluctuated above the *RMSF*_*avg*_. Strnad and Hadcock, in a site-directed mutagenesis study^18^, reported that D3.32 in TM III is a major player in the binding of Somatostatin to SSTR2. We have observed that D3.32 did not fluctuate a lot and stayed around an *RMSF* of ~ 0.1 nm, suggesting the fact that there is no major structural flexibility in the residue conformation. Also, the residue consistently formed H-bonding with the ligands, more often than the other residues. Liapakis et al., in a study to identify the ligand binding determinants, identified that ECL3 and the adjacent TM-spanning regions consist of residues that are crucial for binding of ligands to SSTR2. Particularly, they have reported that a stretch of residues starting from 294 to 297 (FDFV) is important in determining SSTR2 ligand activity. They have also mentioned that the presence of a single hydroxyl group in the ligand can substantially increase the activity of the ligand. Also, it is important to note that the elements present in SSTR2 TM III and IV are responsible for the high affinity of hexapeptide and octapeptide analogs (like SRIF) to SSTR2 over SSTR1. Figure 4 represent the snapshots of the compound **42** inside the SSTR2 binding site over the simulation time. It can be observed that the position of the ligand was stable over time. Parry et al.^19^ reported the importance of the DRY motif (I2.43, D3.49 and R3.50) and found that the mutation of I2.43 resulted in the complete inability of ligand binding. Whereas mutation of the residues D3.49 and R3.50 showed different protein expression levels. We have observed that the DRY residues were stable over the simulation time, scoring *RMSF* values well below the average in both holo and apo forms. This observation suggests that the binding of ligands with the protein did not affect the conformation of these residues.

### Analyzing the pattern of hydrogen bond formation by the ligands

**Figure 5:**
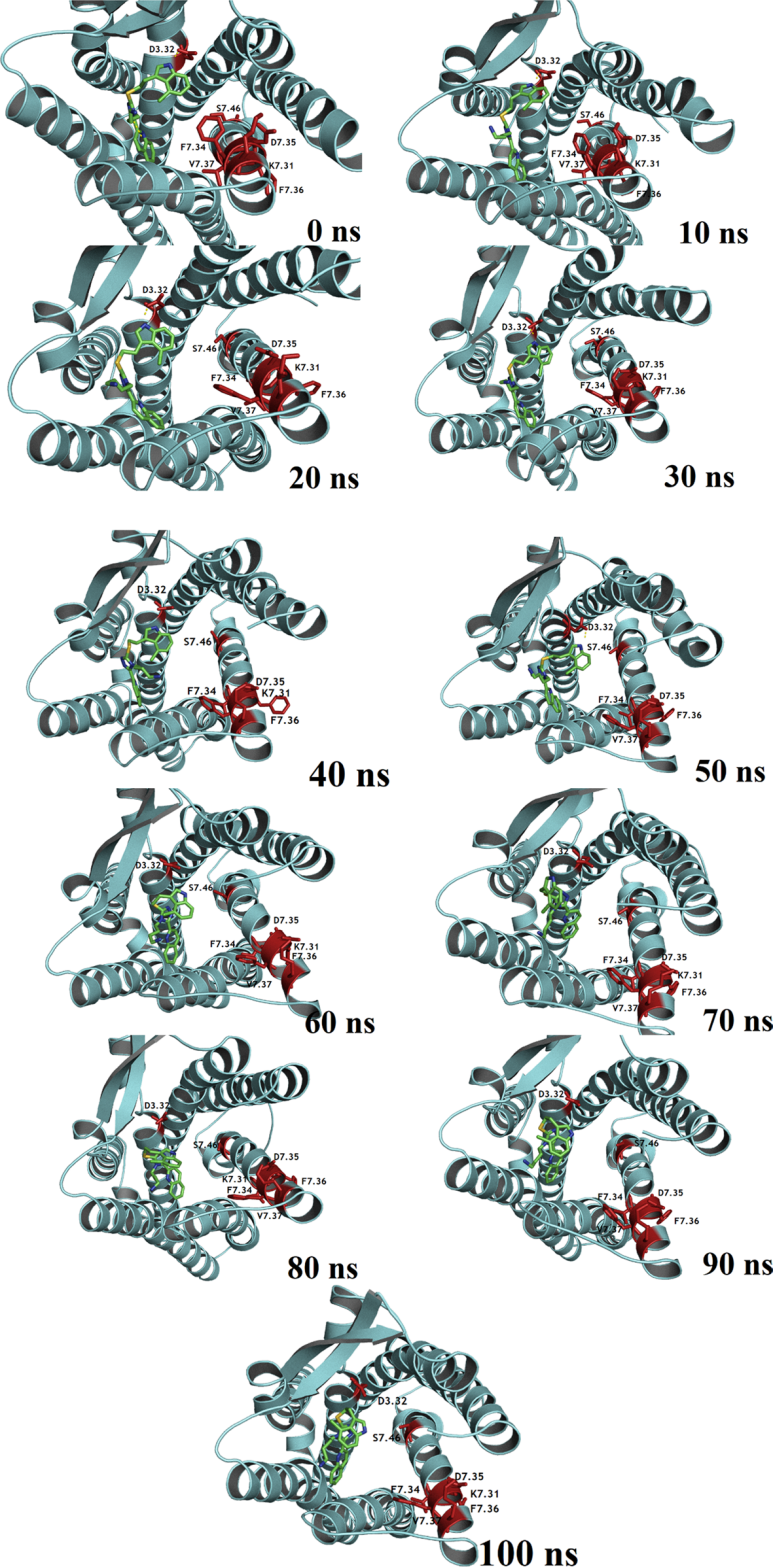
Time evolution of ligand bioactive conformation of compound 46 inside SSTR2 binding site. Figure generated using PyMOL 1.3 (http://www.pymol.org).

**Figure 6:**
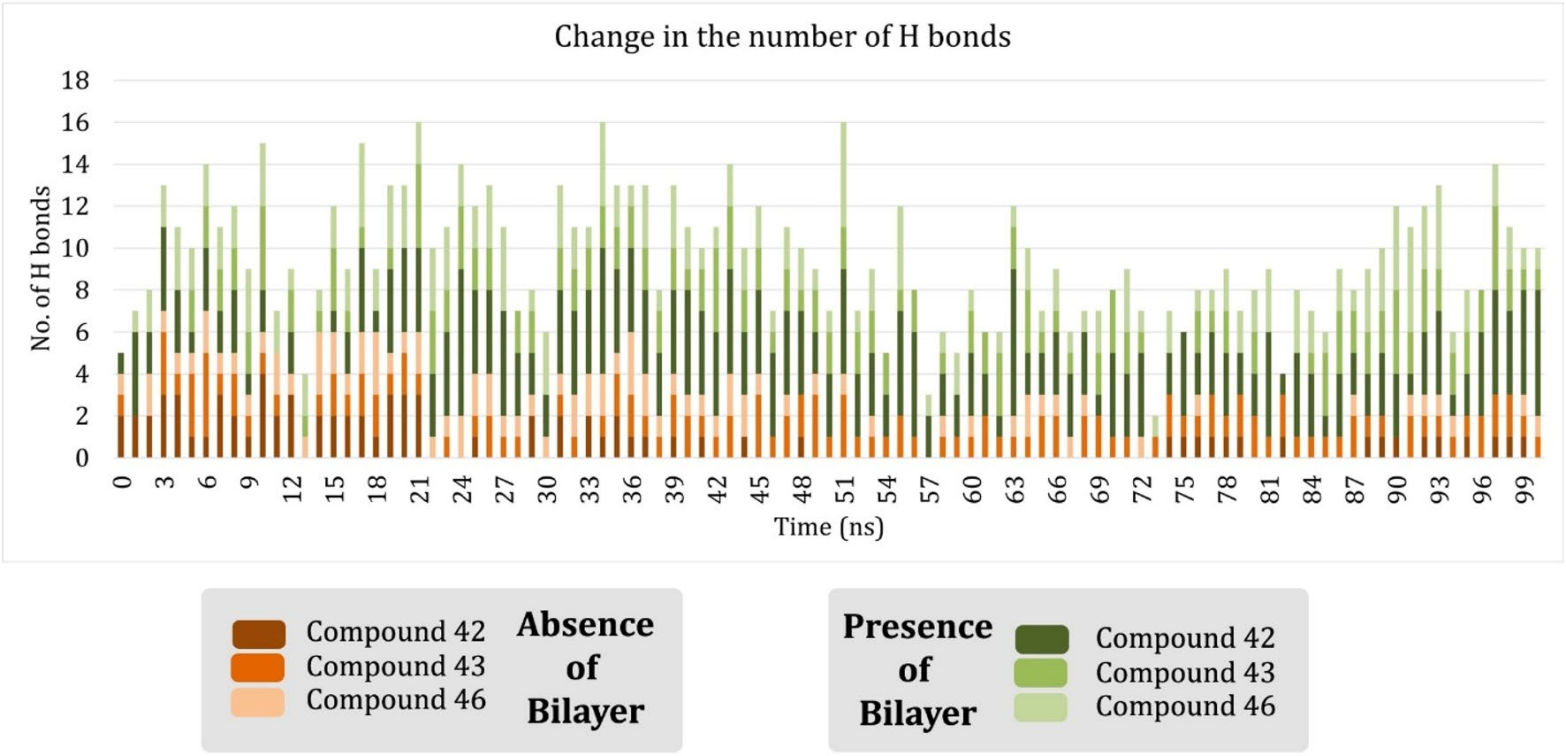
Number of Hydrogen bonds formed by SSTR2 ligands over time in presence and absence of the lipid bilayer. Number of H-bonds formed over time is notably more in the presence of lipids than in the absence of lipids.

After observing the H-bonding pattern of the ligand, we have noted that the experimentally reported residues mentioned in the literature are the major players in H-bonding. To validate this claim, we observed the H-bonding pattern of **43** and **46** over simulation which strengthened the inference that the said residues are crucial in the formation of H-bond. Figure 5 represent the bioactive conformation of **46** during the simulation at different time periods and the different H-bonds formed at the time. On observation, the importance of the residue D3.32 in determining the stability of the SSTR2 ligand was clearly understood. Not only the ligand stayed in the vicinity of the residue but also the residue formed H-bond with the ligand consistently, and it was observed that it was the lone-H-bond-forming-residue at most of the time. A similar H-bonding pattern was observed with compound **43,** where residue D3.32 formed H-bond with the compound more frequently than the other residues. Figure S4 represent the bioactive conformation of compound 43 at various time during the simulation. From the snapshots, we could observe the frequent appearance of D3.32 in H-bond formation with the compound. Other important residues like K7.31, F7.34, D7.35, F7.36, V7.37, and S7.46, which are part of the proposed binding site, formed H-bond scarcely with the ligands. The reason may be the absence of any H-donor group at the position of the ligand which is adjacent to these residues. Hence, an H-donor group at that position will greatly increase the activity of the SSTR2 ligand. The number of hydrogen bonds generated by the ligands with the protein was plotted over time, and we have observed that the SSTR2 ligands formed more hydrogen bonds than the SSTR1 ligands^10^. Compounds **42**, **43** and **46**, formed 1.62, 1.88, and 2 H-bonds respectively with the SSTR2 homology model on an average. The number of hydrogen bonds gradually increased over time, indicating the stability of these molecules inside the predicted binding site. The plot on the number of hydrogen bonds developed by SSTR2 ligands over time is represented in the Fig. 6. On comparing the results with the systems without the bilayer, we observed that the binding site residues formed more H-bonds, which thereby resulted in a more active and stable conformation of the ligands inside the binding site.

### Analyzing the terminal domains

Structural details about the transmembrane domains of GPCR proteins are well characterized and reported by various crystallographic studies, increasing their resolution with every study. Conformational changes that occur during GPCR protein activation are also well documented. However, the extracellular and intracellular domains of GPCRs remain to be explored in a large scale as there is an increase in reports on evidence of their participation in GPCR signaling mechanisms^20^. These structural domains are key contributors to the ability of GPCR to interact with their respective ligands and further in the activation of signaling cascades. One of the main problems in characterizing these domains is due to the exhibition of higher dynamics in comparison with transmembrane domains. Studies regarding the interaction of extra-membranous domains of GPCR with the lipid bilayer is an upcoming trend that can greatly aid in solving the mysteries regarding GPCR structure and function. During crystallization of GPCR proteins, these regions were mostly truncated and if not, it was difficult to resolve them using crystallography and NMR^21–23^. Hence, it is crucial to characterize these regions as they are known to be involved in ligand binding and coupling with G-protein^24,25^. There are studies carried out to relate the role of extracellular domains of SSTRs and their role on SSTR activation and signaling^26–29^.

**Figure 7:**
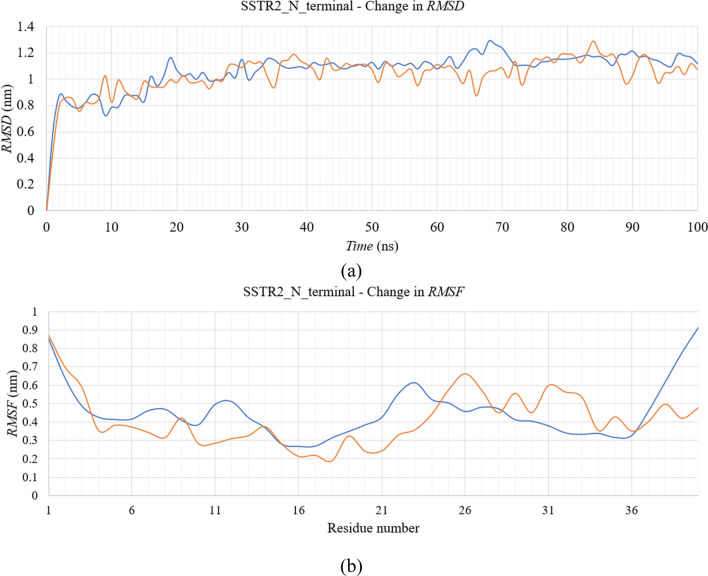
Time evolution of (**a**) *RMSD* and (**b**) *RMSF* of SSTR2 N-terminal domain in the presence or absence of the lipid bilayer. Systems with presence and absence of DPPC bilayer are represented as blue and orange lines, respectively.

**Figure 8:**
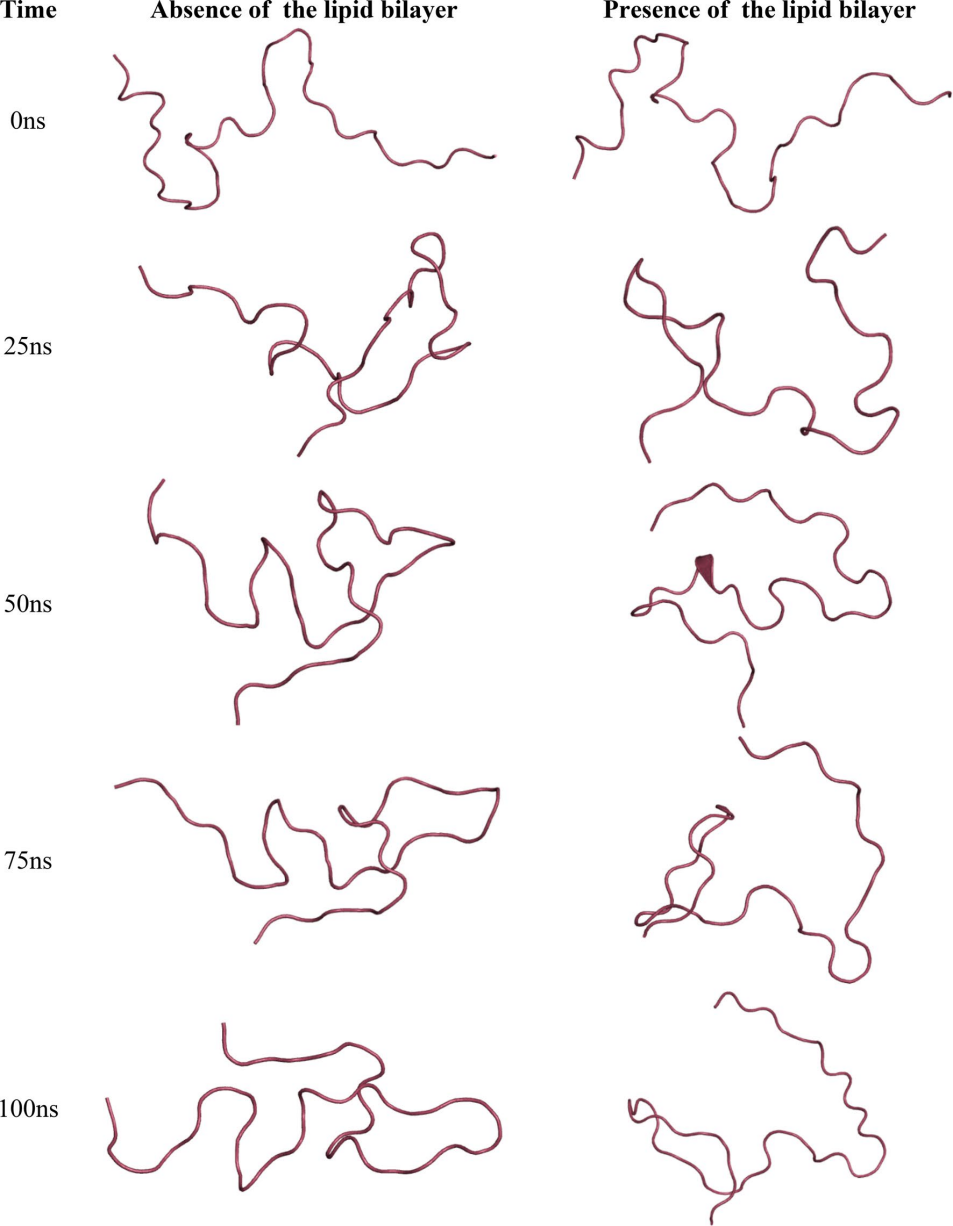
Simulation time-based progression of SSTR2 N-terminal domains in the presence or absence of the lipid bilayer. Figure generated using PyMOL 1.3 (http://www.pymol.org).

**Figure 9:**
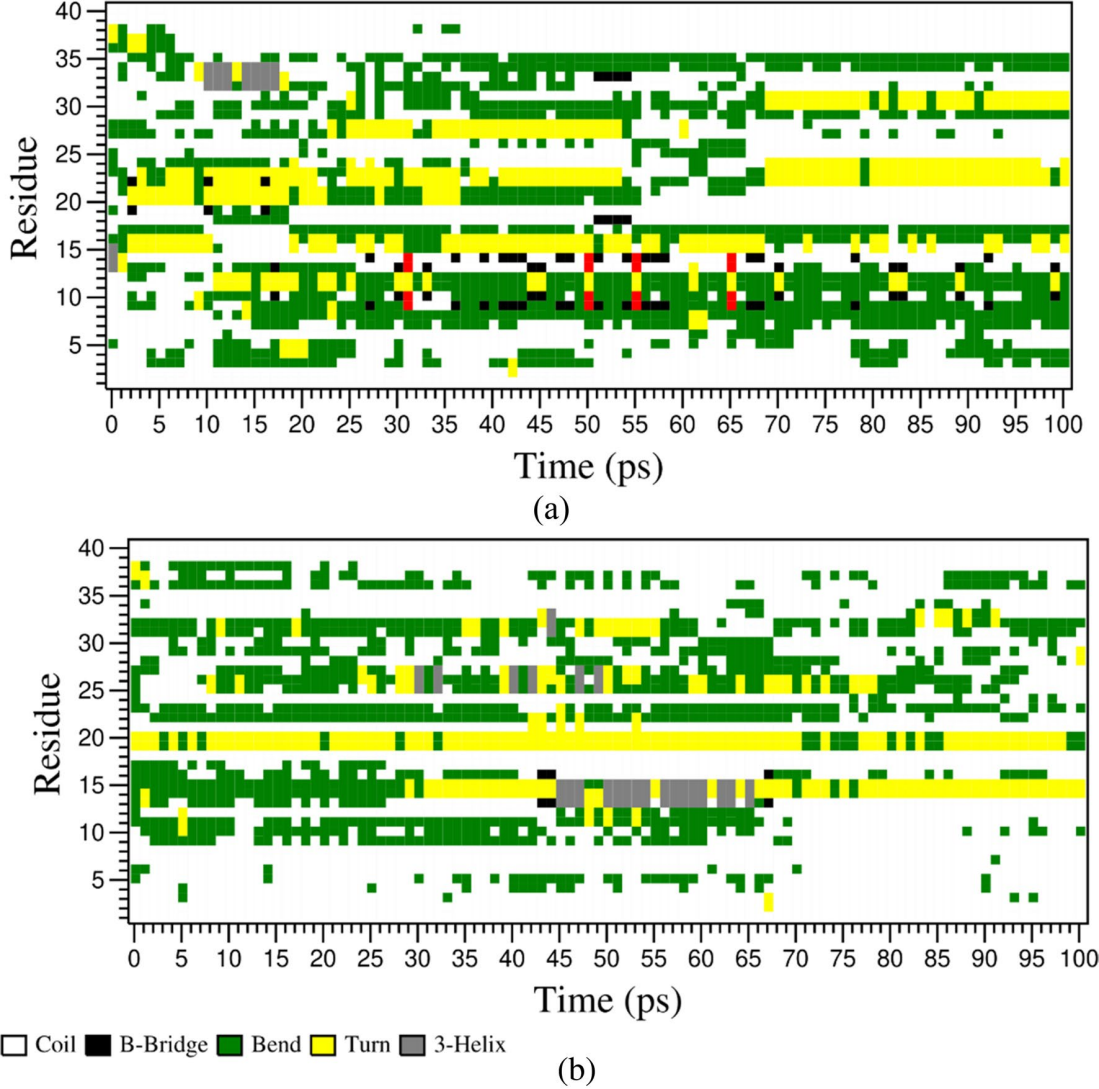
Change in secondary structure elements of SSTR2 N-terminal in the presence (**a**) or absence (**b**) of DPPC bilayer over simulation time.

The three-dimensional structures of N- and C-terminal domains of each subtype are extracted from their respective apo structures. Molecular simulation dynamics for the terminal domains were performed in the presence and absence of the DPPC bilayer. MD trajectories from the simulation runs were obtained and analyzed. In the case of the N-terminal domain, we observed a similar trend of change in *RMSD* in both the systems, with and without the lipid bilayer (Fig. 7). Both the systems equilibrated around ~ 1 nm, after an initial spike from 0 to 1 nm. After analyzing the change in RMSF of the residues over time, we have observed that these residues fluctuated a lot lesser than the N-terminal residues of the other subtypes. All the residues fluctuated below ~ 1 ns, which is considerably lower. However, the differences between RMSF of N-terminal residues in the two systems were minimal. Snapshots of the complexes at different time frames were taken to analyze the time-dependent change in the protein conformation (Fig. 8) and for a better understanding, the snaps were compared with the DSSP plots developed for both the systems (Fig. 9). Residues 1 to 10 and residues 25 to 35 formed similar structural elements, bends, at times over the simulation. In the case of the residues 11 to 20, bends and turns were formed intermittently in both systems. The difference being that these residues form β-helices and β-bridges at times in the presence or absence of the bilayer, respectively. Residues 35 to 40 did not form any secondary structural element after ~ 5 ns in the presence of the bilayer, however, the formation of intermittent bends was evident in the absence of the bilayer. Overall, there were no considerable differences in the structural conformation of SSTR2 N-terminal domain between the two systems.

**Figure 10:**
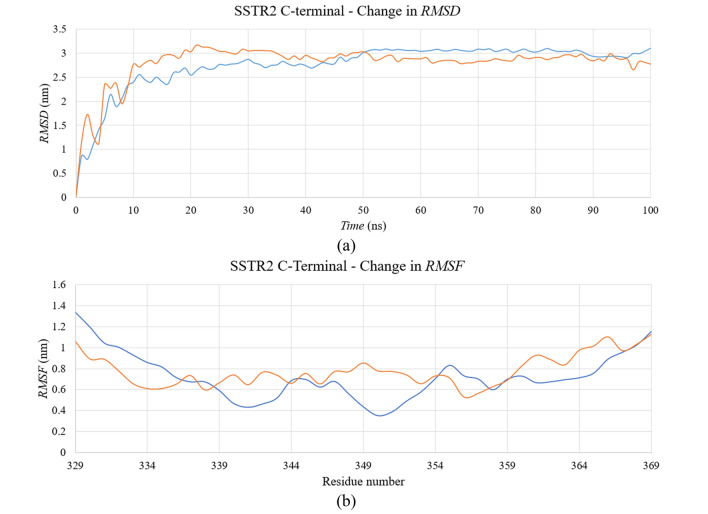
Time evolution of (**a**) RMSD and (**b**) RMSF of SSTR2 C-terminal domain. Systems with presence and absence of DPPC bilayer are represented as blue and orange lines, respectively. Figure generated using PyMOL 1.3 (http://www.pymol.org).

**Figure 11:**
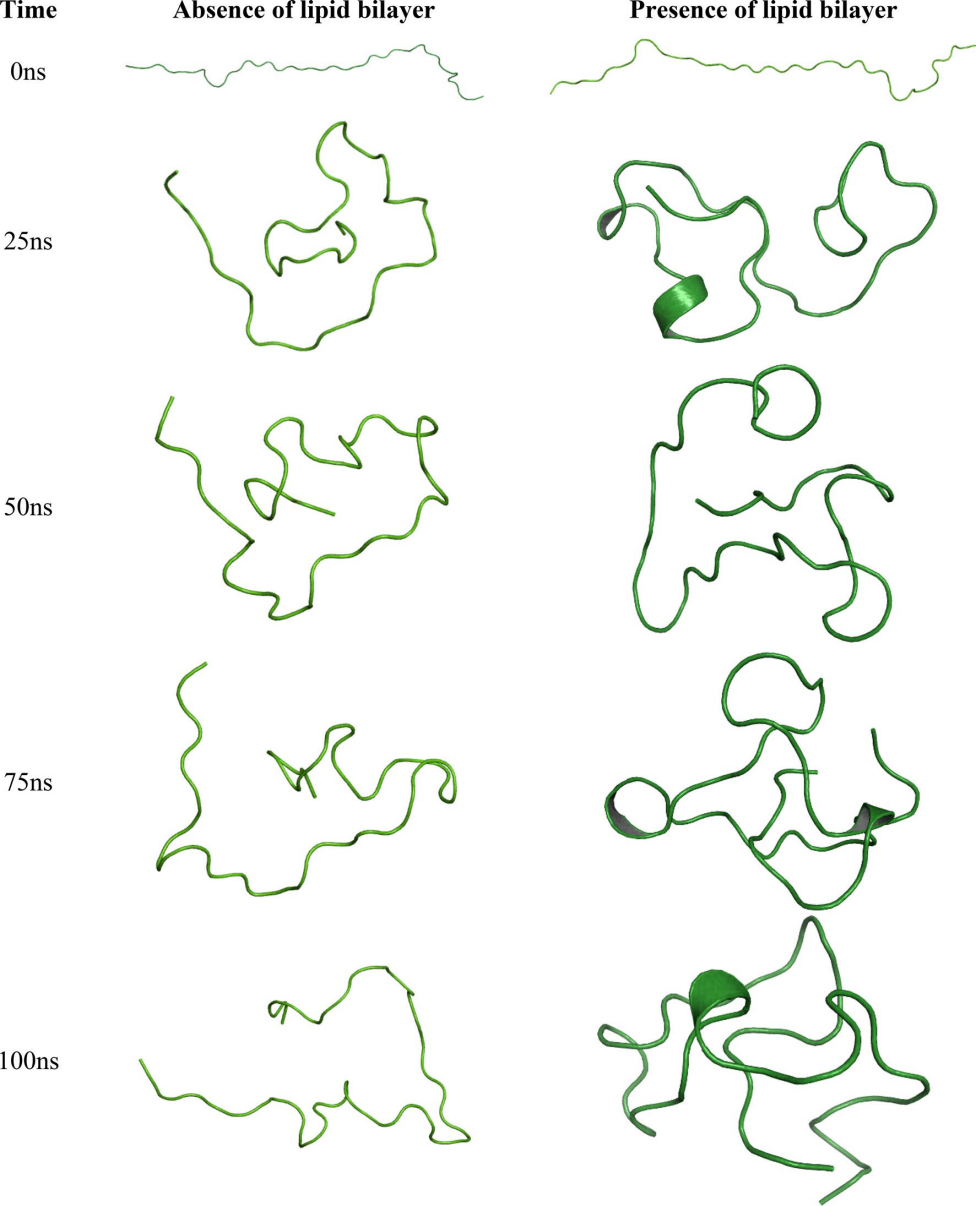
Simulation time-based progression of SSTR2 C-terminal domain in the presence and absence of lipid bilayer. The domain formed more secondary structure elements in the presence of lipids.

**Figure 12:**
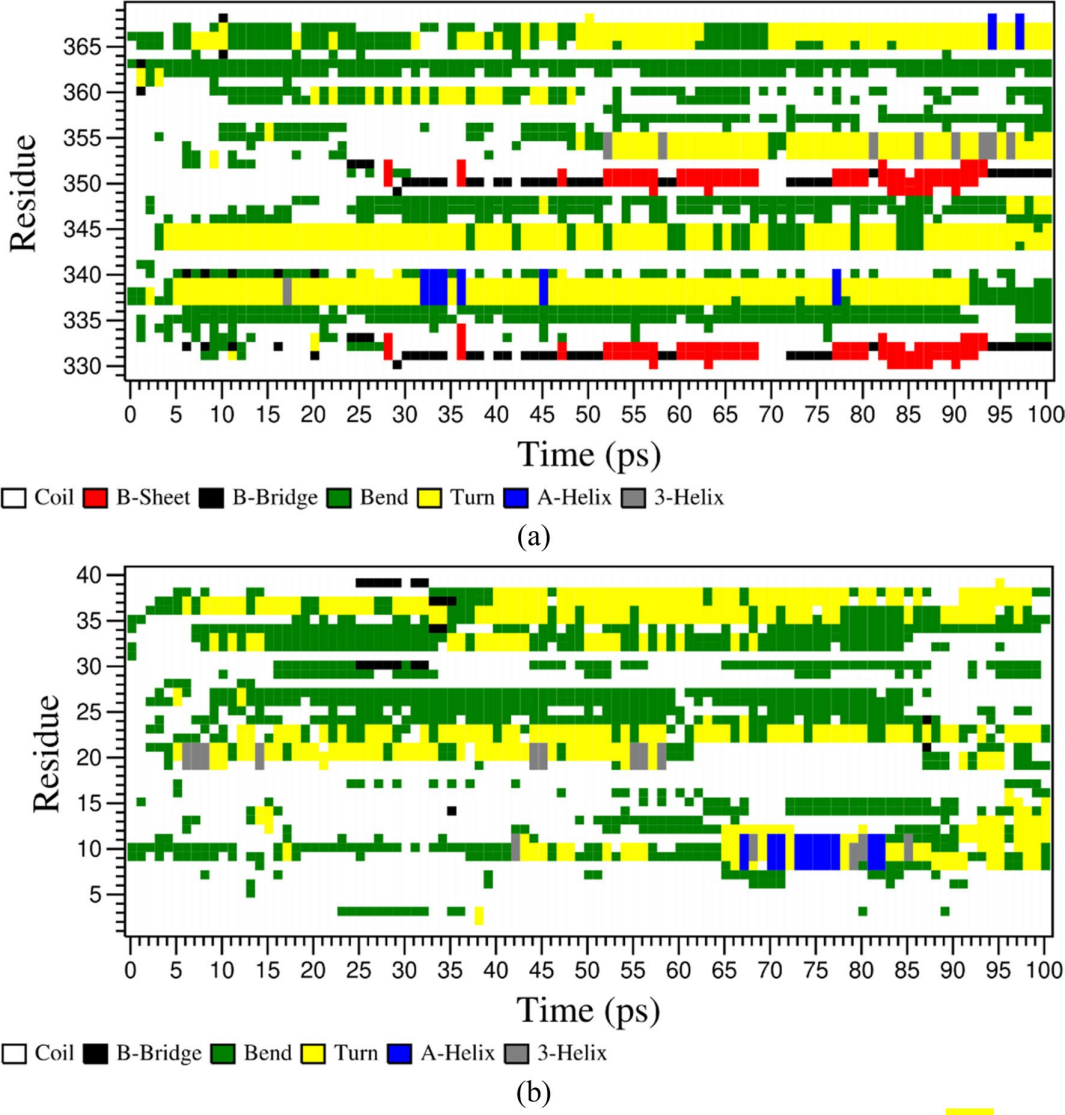
Change in secondary structure elements of SSTR2 C-terminal in the presence (**a**) and in the absence (**b**) of DPPC bilayer over simulation time. The domain formed more β-sheets in presence of lipid molecules during the simulation.

In the case of the C-terminal domain, we did not observe considerable differences with the *RMSD* of two systems, in the presence or absence of lipid bilayer (Fig. 10). However, both the systems equilibrated around ~ 3 nm after 20 ns, which is significantly higher, in comparison with the other subtypes. Similar is the case with the change in *RMSF* backbone Cα atoms of the residues. At no point of the simulation, none of the residues showed > 0.4 nm difference between the two systems. As there is no clear difference observed between the two systems based on *RMSD* and *RMSF*, we extracted the domain coordinates at different timestamps (0 ns, 25 ns, 50 ns, 75 ns and 100 ns) to better understand the differences between the two systems. In Fig. 11, the structural conformations of the SSTR2 C-terminal domain at different time frames are represented in the form of cartoon representation. In a study by Zitzer et al.^30^, it was reported that the SSTR2 C-terminus is important in interacting with multidomain proteins present in the human brain. In the study, the potentially interacting proteins with SSTR2 were identified by using the C-terminus of SSTR2 as a bait in a yeast two-hybrid screen. The isoleucine present at the end of the SSTR2 C-terminus was replaced by seven amino acid residues and was cloned into a bait vector. The study revealed an important observation that the modified C-terminus region showed no growth on His-deficient media, whereas the SSTR2 construct showed the highest number of colonies. This observation signifies the importance of the folding of C-terminal residues in the functioning of SSTR2. Han et al.^31^ studied the importance of SSTR2 cytoplasmic C-terminal residue beyond amino acid 314 in ligand binding. The findings of the study suggested that SSTR2 with a deletion of amino acids beyond would be signal deficient, thereby proving the importance of C-terminus in ligand binding. From our study, we observed that the presence of lipids largely influences the folding of C-terminal residues. To understand more about the difference in folding between two systems, we analyzed the secondary structural elements formed by these residues over time in both the systems and observed the difference between the two systems. Additionally, secondary structure elements formed by the residues over time is compared with the extracted snapshots of the domain at different time frames. Figure 12 represents the plot of *DSSP* for both the systems over simulated time. Residues 330 to 335 formed no secondary structural elements in absence of the bilayer, while they formed β-sheets and bridges in the presence of a bilayer. Similarly, residues 336 to 340 did not form any elements in absence of the bilayer but formed more turns in presence of the lipids. Residues 350 to 355 formed bends and turns in the absence of DPPC which are transformed into β-turns and β-helices in the presence of DPPC. These residues consist of the serine cluster (Ser 341/343) and threonine clusters (Thr 353/354) which are important for desensitization, receptor internalization, and β-arrestin binding^32–34^. α-helices are seen at certain points of simulations in both systems. The differences in the folding displayed by the domain in the presence and absence of lipid molecules might be useful in understanding the secondary structural elements that are responsible for the functioning of SSTR2.

## Discussion

This study is a part of a broad study to understand the structural features responsible for the bioactivity of the Somatostatin receptors^10–12^. In this study, we have analyzed the conformational dynamics of different structural domains of SSTR2 in the presence and absence of the lipid molecules. The results of this study were correlated with the previously reported structural characterization studies of SSTR2, which facilitated a better understanding of the SSTR2 structure. By comparing the results of both the studies, we have observed the importance of lipid molecules in the secondary structure formation of different domains of SSTR2. Also, we studied the important SSTR2 residues taking part in the formation of receptor-ligand complex. After validating the reliability of simulated systems using different MD analysis techniques, we studied the MD trajectories to understand more about their structural dynamics over time. The most important finding we have observed is the importance of the residue D3.32 in determining the stability of SSTR2 ligands with the receptor. It correlates well with the previously reported SSTR2 structural characterization study^11^. In both studies, D3.32 formed hydrogen bonds more often than the other residues. We observed a tendency of the ligand molecules to move deep inside the binding pocket to form an H-bond with the D3.32 residue. On analyzing the simulation trajectories, the number of H-bonds formed by the ligands with D3.32, notably, increased over time. We observed similar results from molecular docking of different SSTR2 ligand datasets, native peptides like SRIF-14, SRIF-28, and somatostatin analogues^11^. It is important to note that, based on a site-directed mutagenesis study, Strnad and Hadcock identified that D3.32 present in the TM III is a key player in SSTR2-somatostatin binding^18^. Another important observation is that an SSTR2 ligand with an H-bond donor group at R′ position can significantly increase the activity of the molecule. From our previous report on 3D-QSAR studies on SSTR2 ligands^11^, we observed that the H-donor at R_1_ substitution position increases the bioactivity of the molecules. Also, in that study, we analyzed the dataset using DFT-based descriptors^11^ and identified that molecules showing high electronegativity had better activity. In addition to that, we observed that a hydrophobic group at R2, like sulfur increases the activity. Contour-Galcéra et al.^35^ in the SAR study of 3-Thio-1,2,4-triazoles as novel somatostatin sst2 agonists reported that the hydrophobic groups such as butylamine at R2 and indole at R3 increases the molecular activity. Hence, in order to develop novel SSTR2 agonists with better activity and high SSTR2 selectivity, it is suggested for the compounds to have a non-bulky, H-donor substitution at R1, hydrophobic groups at R2 and R3.

While there are many studies available on the structural biology of transmembrane helical domains of GPCR proteins, the number of studies available on the extracellular domains of GPCRs lags a lot. Due to the higher dynamics exhibited by these domains, it becomes difficult to crystallize these regions and, to study. Studying the conformational dynamics of the N- and C-terminal regions of SSTR2 will aid in understanding their importance in SSTR2 functioning, hence, we performed molecular simulations of these regions in the presence and absence of the lipid bilayer. We studied the changes in the secondary structural elements formed by the different combinations of residues in these regions over time. We observed a change in the formation of secondary structure elements in the presence of a lipid bilayer. From our study, we observed that the lipid molecules played a major role in regulating the folding of the C-terminal residues. In presence of lipid molecules, the frequent appearances of β-sheets suggested the importance of lipid molecules in the folding. Our results confirm to the previous reports of Zitzer et al. and Han et al. on the importance of C-terminal residues in SSTR2 functioning. Despite these observations, a study by Schwartkop^36^ reported an interesting result related to the importance of the SSTR2 C-terminus. In this study, different C-terminal deletion constructs were investigated for their ability to undergo agonist-dependent internalization. While the constructs lacking 10, 30 and 44 amino acids in the C-terminus displayed SRIF14-dependent internalization, a construct lacking 20 amino acids was detected independently of the agonist^36^. A recent study by Olsen et al.^37^ demonstrated that the endosomal trafficking of SSTR2 is influenced by various regulatory mechanisms controlled by the C-terminus. Hence, it is clear that, despite the presence of various reports on SSTR2 functioning, the complexity of the signaling pathway warrants further studies on the different important residues involved in SSTR2-ligand binding. Structural biology studies targeting these residues could reveal more about the importance of these residues in the bioactivity of SSTR2.

## Materials and methods

Software like MODELLER and Sybyl-X 2.1 were used to perform protein modeling, molecular docking, and ligand preparation, installed in a Windows environment. For performing molecular simulations, we used GROMACS (v. 2016.3) installed in a Linux platform.

### Homology modeling and model validation

As there are no crystal structures of SSTR2 is available in the protein data bank (PDB)^38^, the three dimensional (3D) structure of the receptor was modeled using homology modeling. When only the sequence information available about a receptor is available, reliable 3D models of proteins can be provided by homology modeling^39^. The information about the modeling platforms used, the number of generated models, model validation calculations, and selected 3D models were present in a previous SSTR2 structural characterization study reported by our group^11^.

### Ligand dataset selection, binding site prediction and molecular docking

The information regarding the binding site prediction tools used to predict the probable SSTR2 binding is previously reported^11^. Molecular docking of different SSTR2 ligands from datasets reported by Zhou et al.^40^ and Contour-Galcéra et al.^35^ was followed. The ligand molecules used in the study along with their activity values are reported in Table S1. Surflex docking algorithm present in Sybyl-X 2.1 was used to perform molecular docking. In this study, we used SSTR2-ligand complexes with highly active compounds like **42**, **43**, and **46** to perform molecular simulations. The pK_i_ values of compounds **42**, **43**, and **46** are 7.92, 8.74, 8.25, respectively.

### Molecular dynamics (MD) simulation

For the cell-to-cell transport of different substrates via the cell membrane, the lipid bilayer acts as a gatekeeper. This transport process is important for the different biological processes like signaling. In order to study the role of lipid membrane on the above aspect, molecular dynamics can be a useful tool. SSTR is a membrane protein, studying its conformational diversities using molecular dynamics adds to the knowledge on the SSTR-related signaling mechanism. Hence, we studied the dynamics of SSTR2 in presence and absence of lipid bilayer. As mentioned already, we selected the SSTR2-ligand complexes with SSTR2 ligands **42**, **43,** and **46** to study their conformational dynamics over time using molecular dynamics. To perform the simulations, we used the GROMACS molecular simulation software (v. 2016.3). We used CHARMM-GUI^41^, a web-based platform, to embed the protein–ligand complex in a lipid bilayer made up of DPPC molecules. The platform helped in generating the different input files required to perform simulations using GROMACS^42,43^.

While constructing a membrane protein-lipid bilayer complex, it is crucial to orient the protein in a way that the non-polar lipid tails of the membrane bilayer with the hydrophobic region of the protein. Hence, to arrange the said regions, we used the Orientations of Proteins in Membranes (OPM) database (https://opm.phar.umich.edu/). The database contains spatial coordinates of the membrane proteins reported in the PDB, that are oriented to the hydrophobic core of the membrane bilayer. As SSTR2 does not have a solved structure in the PDB, we used the OPM coordinates of one of the modeling templates (5C1M). After downloading the OPM of 5C1M, we aligned the selected SSTR2 homology model with the OPM coordinates and used the model for simulation. 5C1M is the crystal structure of the active mu-opioid receptor, which is a GPCR protein and has a ~ 40% sequence identity with SSTR2. *Membrane builder*^41,44^ was used to construct the lipid bilayer, with 200 DPPC molecules each on the top and bottom layer. DPPC molecule is one of the most extensively studied phospholipids and widely used in the simulation of membrane-protein systems. In both the top and bottom layers, the water thickness was maintained at 20.0 Å. The replacement method was used to construct the different components of the system such as the lipid bilayer, bulk water, and counter ions. After constructing the system, the overall simulation protocol including system optimization, equilibration, and simulation run was performed in 6 steps^41^. At every step, it was ascertained that the constructed system was equilibrated gradually. The steepest descent convergence method was used to minimize the system energy. In each step, different restraint forces like harmonic, repulsive planar, and planar restraints were minimized. We performed the simulations in an all-atom CHARMM36m force field.

## Supplementary Information

Supplementary Information
