# Supplementary material for: Understanding the influence of lipid bilayers and ligand molecules in determining the conformational dynamics of somatostatin receptor 2
Source: Sci Rep. 2021 Apr 7;11:7677. doi: 10.1038/s41598-021-87422-5 (PMC8027056; doi:10.1038/s41598-021-87422-5)
Supplement: Supplementary file 1 — Supplementary Information [file 41598_2021_87422_MOESM1_ESM.pdf]

Understanding the influence of lipid bilayers and ligand molecules in determining the conformational dynamics of Somatostatin receptor 2

*Santhosh Kumar Nagarajan[1], Sathya Babu[1], Seema A. Kulkarni[2], Aanand Vadivelu[1], Panneer Devaraju[3], Honglae Sohn[4#], Thirumurthy Madhavan[1]**

[1] Computational Biology Lab, Department of Genetic Engineering, School of Bioengineering, SRM Institute of Science and Technology, SRM Nagar, Kattankulathur, Chennai 603203, India.

[2] Department of Food and Process Engineering, School of Bioengineering, SRM Institute of Science and Technology, SRM Nagar, Kattankulathur, Chennai 603203, India.

[3] Unit of Vector Biology & Control, ICMR-Vector Control Research Centre, Indian Council of Medical Research (ICMR), Puducherry, India

[4] Department of Chemistry and Department of Carbon Materials, Chosun University, Gwangju, South Korea.

[*] Corresponding author

Email: thiru.murthyunom@gmail.com, thirumurthy.m@ktr.srmuniv.ac.in
Address: Department of Genetic Engineering, School of Bioengineering, SRM Institute of Science and Technology, SRM Nagar, Kattankulathur, Chennai-603203, India.
Phone: +91 99445 72918

[#] Co-corresponding author

Email: hsohn@chosun.ac.kr
Address: Department of Chemistry and Department of Carbon Materials, Chosun University, Gwangju, South Korea.
Phone: +82 (010) 5475 – 0163

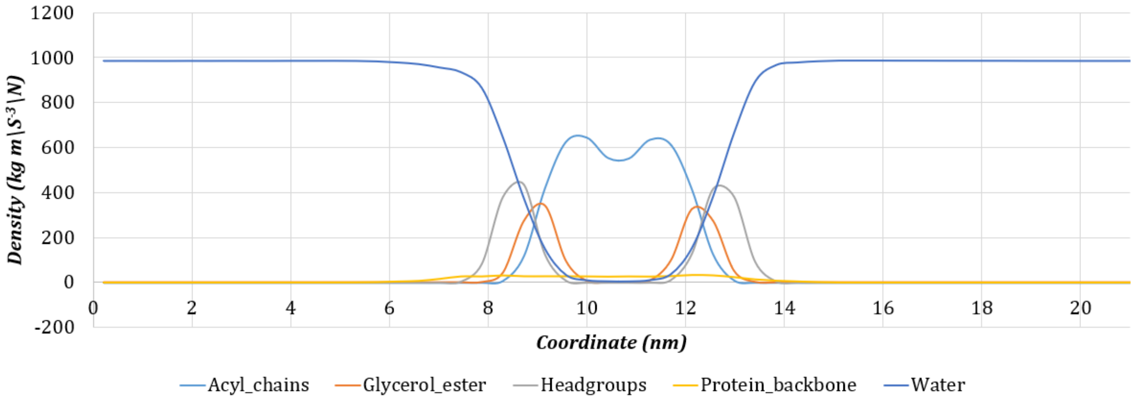

(a)

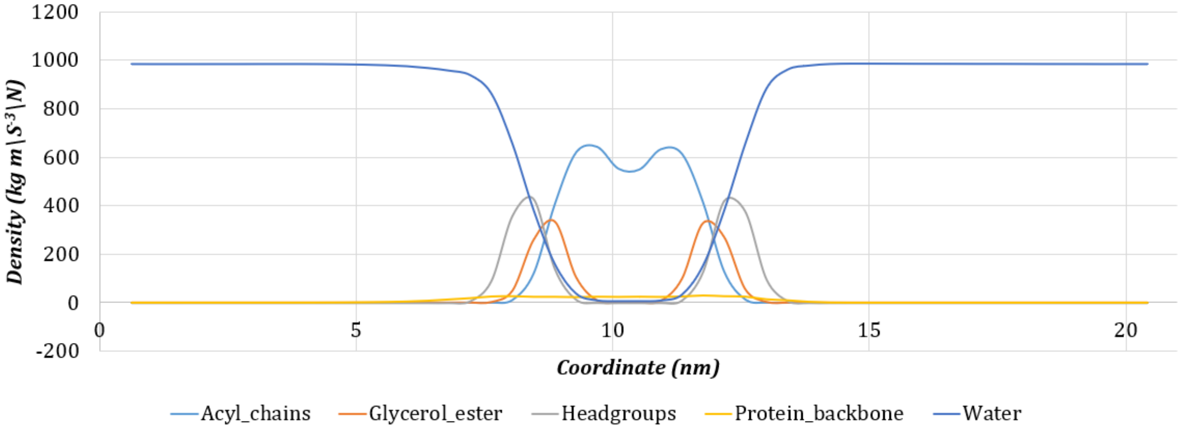

(b)

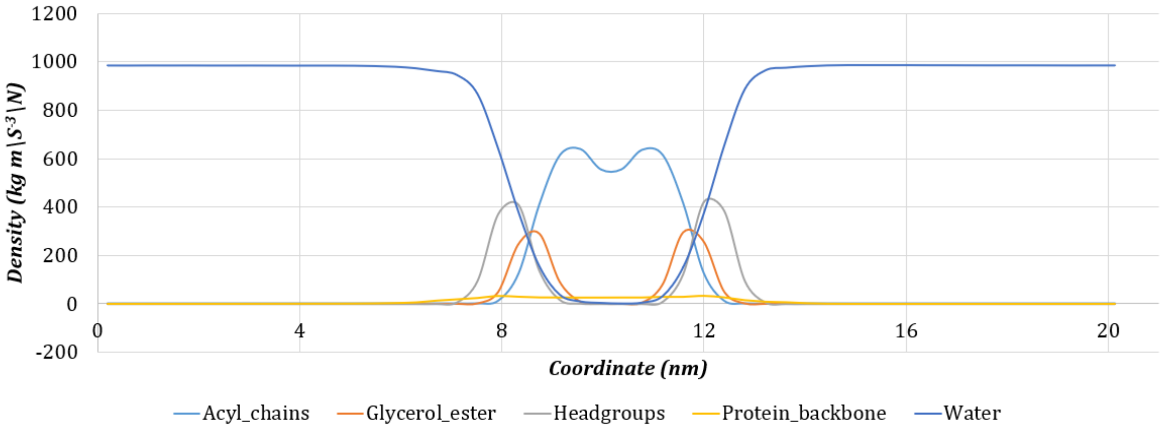

(c)

Figure S1. Average density of different membrane components over the simulation time for SSTR2 systems with compounds (a) 42 (b) 43 (c) 46

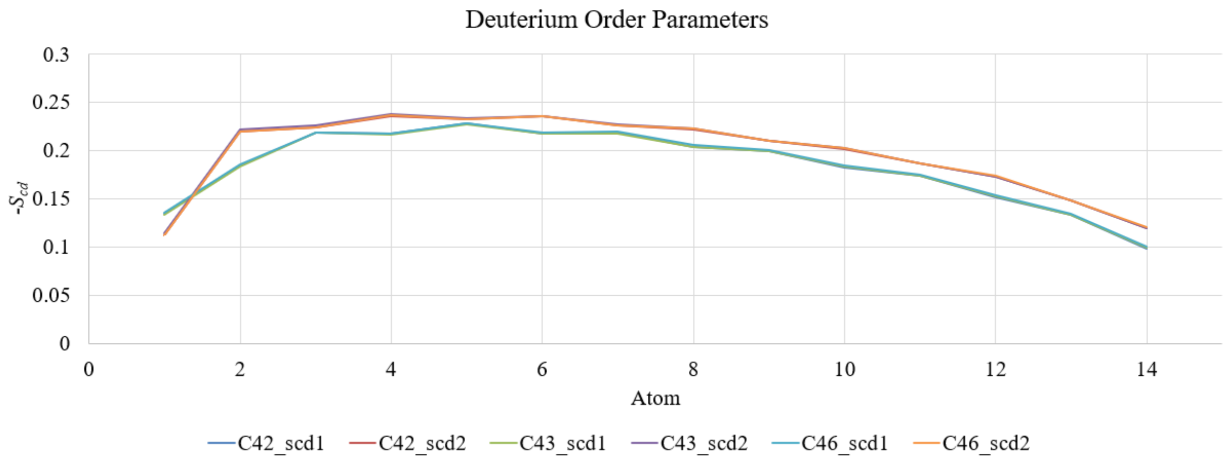

Figure S2. Comparison of deuterium order parameters of each simulated system.

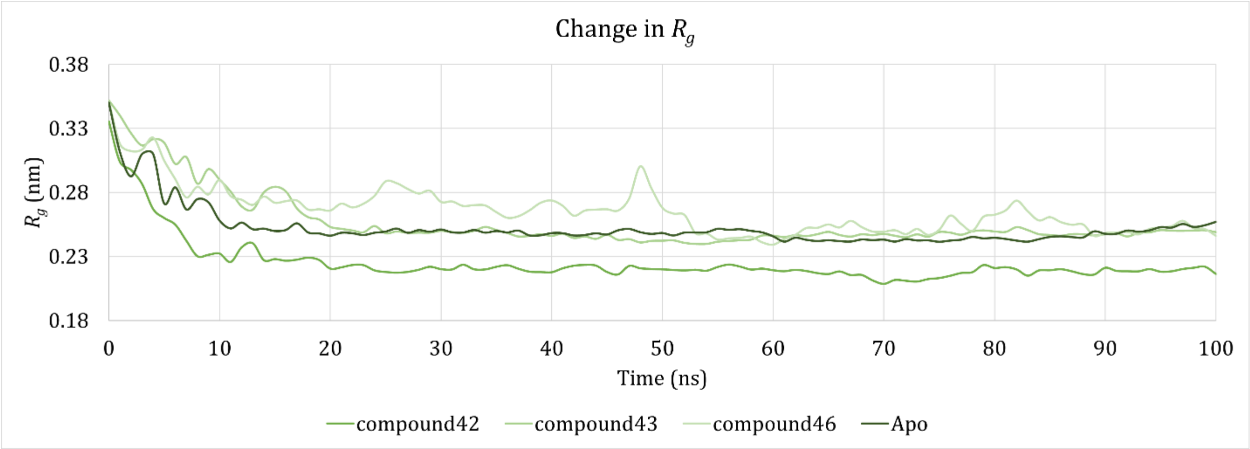

Figure S3. Change in R_g of the backbone Cα atoms of SSTR2 complexes over 100 ns.

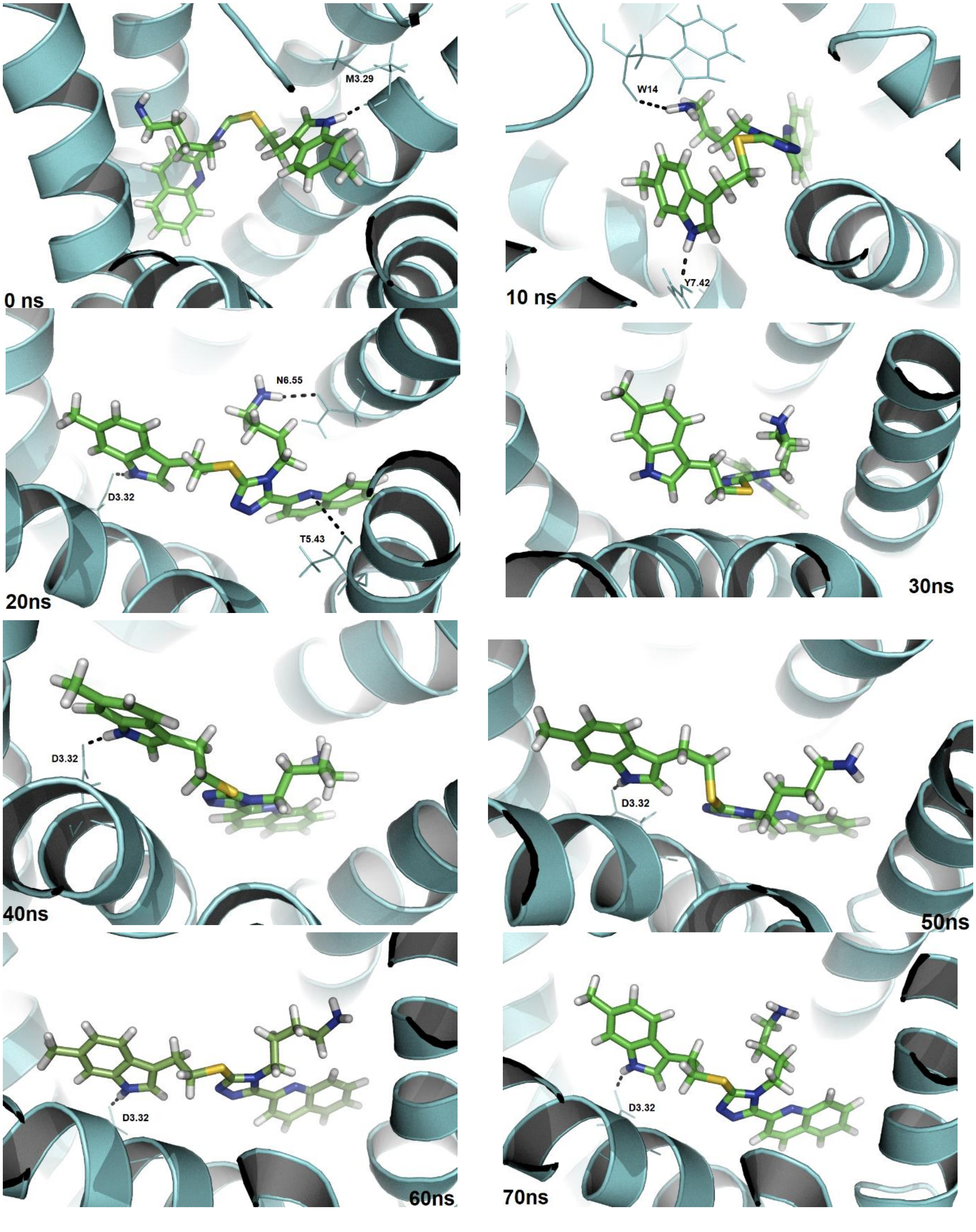

M3.29
0 ns
W14
Y7.42
10 ns
N6.55
D3.32
T5.43
20ns
30ns
D3.32
40ns
D3.32
50ns
D3.32
60ns
D3.32
70ns

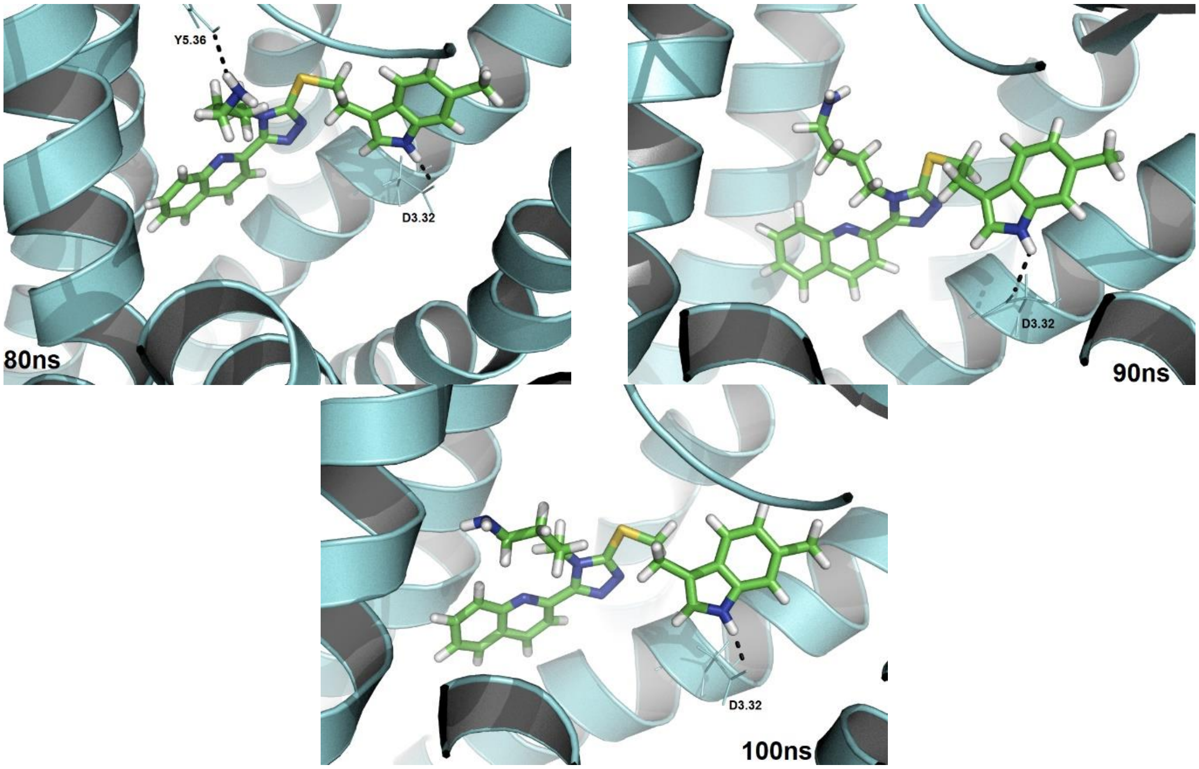

Figure S4. Bioactive conformation of compound 43 inside SSTR2 binding site at various time stamps. From the snapshots, it can be observed that the residue D3.32 formed H-bond more frequently with the compound than other residues. Figure generated using PyMOL 1.3 (http://www.pymol.org).

Table S1: Chemical structures and biological activities of SSTR2 Agonists

Nipecotic and iso-Nipecotic Amides					
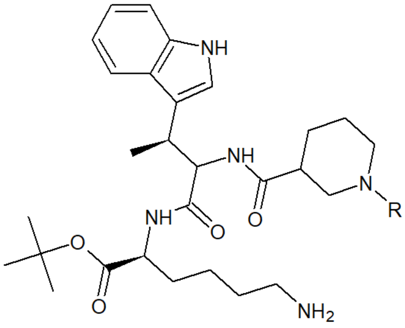					
**Compound**	**R**	***pK**i***	**Compound**	**R**	***pKi***

01	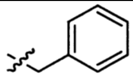	8.3372	16	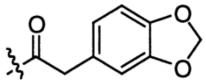	8.0000
02	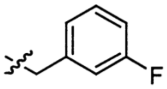	8.3010	17	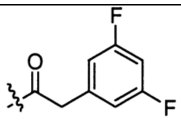	8.6383
03	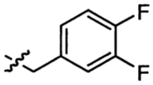	8.5376	18	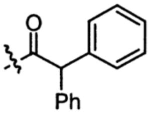	8.3010
04	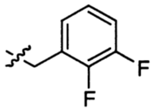	8.2441	19	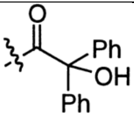	8.5528
05	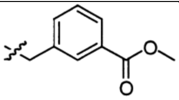	7.9586	20	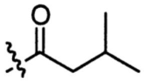	7.6383
06	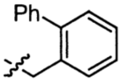	7.6778	21	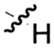	6.7496
07	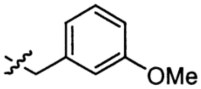	8.0000	22	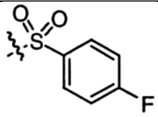	7.9586
08	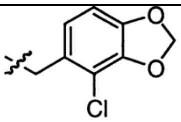	7.9208	23	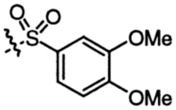	7.6383
09	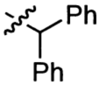	7.5086	24	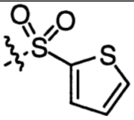	7.8539
10	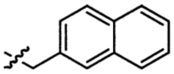	7.7696	25	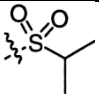	7.4559
11	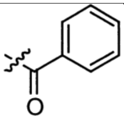	8.0000	26	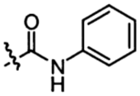	7.9586
12	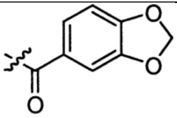	7.4437	27	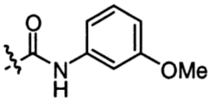	8.0458

13	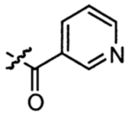	7.0706	28	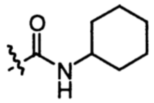	8.1427
14	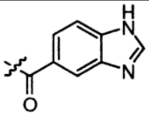	7.0706	29	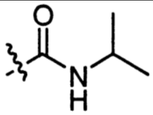	7.0223
15	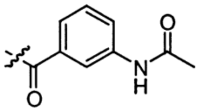	7.3279	30	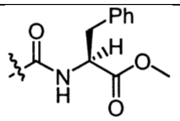	8.0655
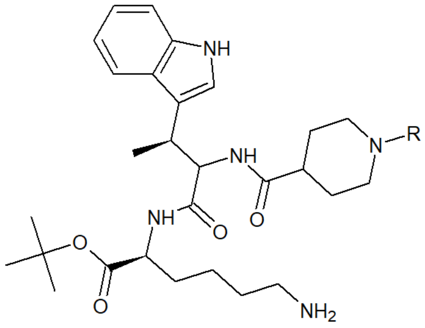					
31	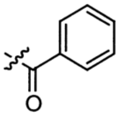	9.3010	33	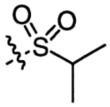	6.9208
32	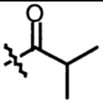	7.8239	34	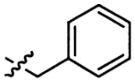	8.6021
**3-Thio-1,2,4-triazoles**					
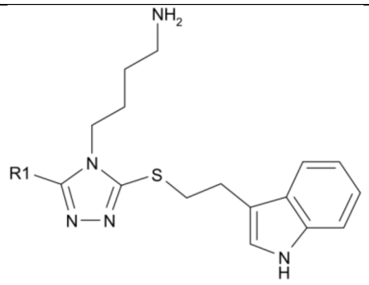					
**Compound**	**R1**	***pKi***	**Compound**	**R1**	***pKi***

35	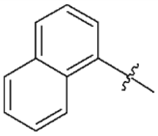	6.8239	39	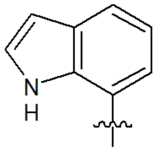	6.6576
36	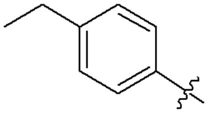	6.3665	40	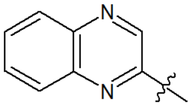	7.4949
37	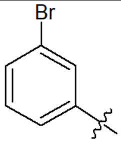	6.6021	41	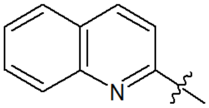	7.9208
38	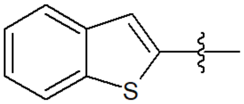	6.7959	42	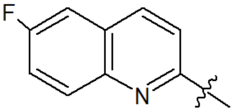	7.9208
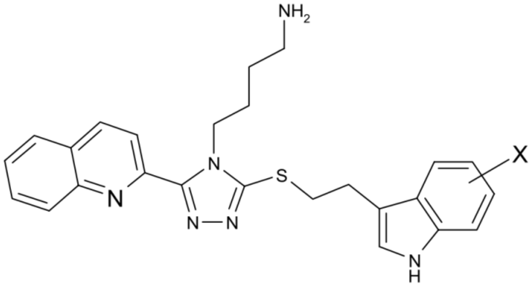					
**Compound**	**X**	***pKi***	**Compound**	**R1**	***pKi***
43	7-Me	8.7447	45	7-Cl	8.3979
44	6-F	8.5686	46	5-Cl	8.2518
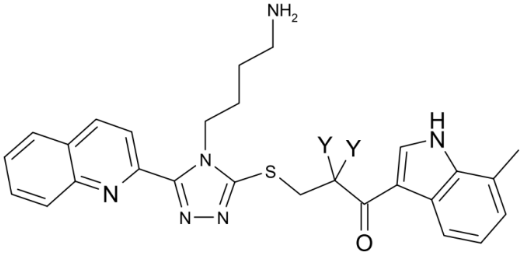					
**Compound**	**Y**	***pKi***	**Compound**	**R1**	***pKi***
47	H	8.6990	48	Me	7.6383
